# Influence of Enzyme-Inducing Antiepileptic Drugs on Trough Level of Imatinib in Glioblastoma Patients

**DOI:** 10.2174/157488408785747656

**Published:** 2008-09

**Authors:** Stefan Pursche, Eberhard Schleyer, Malte von Bonin, Gerhard Ehninger, Samir Mustafa Said, Roland Prondzinsky, Thomas Illmer, Yanfeng Wang, Christian Hosius, Zariana Nikolova, Martin Bornhäuser, Gregor Dresemann

**Affiliations:** 1 Carl Gustav Carus University, Internal Medicine I, Division Hematology and Oncology, Dresden, Germany; 2 Carlvon-Basedow Hospital Merseburg, Internal Medicine II, Division Hematology and Oncology, Merseburg, Germany; 3 Franziskus-Hospital, Internal Medicine, Division Hematology and Oncology, Dulmen, Germany; 4 Novartis Pharmaceuticals Corporation, Florham Park, USA and Nürnberg, Germany

**Keywords:** Imatinib, STI571, CGP74588, EIAED, phenytoin, valproate, carbamazepine, oxcarbazepine, topiramate, lamotrigine, levetiracetam, pharmacokinetics, main metabolite, cytochrome P450, CYP3A4, glioblastoma multiforme, CML.

## Abstract

**Background:**

Imatinib mesylate is used in combination with hydroxyurea (HU) in ongoing clinical phase II studies in recurrent glioblastoma multiforme (GBM). CYP3A4 enzyme-inducing antiepileptic drugs (EIAEDs) like carbamazepine, phenytoin, and oxcarbazepine - as well as non-EIAEDs like valproic acid, levetiracetam, and lamotrigine - are frequently used in patients with GBM. Since CYP3A4 is the major isozyme involved in the metabolism of imatinib, we investigated the influence of EIAEDs on imatinib pharmacokinetics (pk).

**Methods:**

GBM patients received 600 mg imatinib p.o./o.d. in combination with 1.0 g HU p.o./o.d..together with either EIAEDs, non-EIAEDs, or no antiepileptic drug (non-AEDs) comedication. Trough plasma levels of imatinib and its active main metabolite N-desmethyl-imatinib (CGP74588) were determined biweekly in these patients, total 543 samples being collected from 224 patients (up to 6 times / patient). All three groups were compared to each other and with historical pharmacokinetic data obtained from patients with chronic myeloid leukemia (CML).

**Results:**

Mean imatinib trough levels in patients not receiving AEDs ( 1404 ng/ml, CV 64%) and on non-EIAEDs (1374 ng/ml, CV 46%) were comparable with mean imatinib trough levels of the historical control group of CML patients (1400 ng/ml, CV 50%). Mean trough levels of imatinib were reduced up to 2.9-fold (477 ng/ml, CV 70%) in patients treated with EIAEDs. Only slight, but although significant differences were observed in the mean trough level of the metabolite CGP74588 between EIAED-, non-EIAED and no-AED patients, 240 ng/ml (CV 57%) , 351 ng/ml (CV 34%) and 356 ng/ml (CV 52%), respectively. The corresponding mean level for CML patients was 300 ng/ml (CV 50%).

**Conclusion:**

Significant decreases of imatinib and CGP74588 trough levels were observed for patients receiving EIAEDs. The EIAED-induced reduction in trough imatinib levels can be avoided by switching to non-EIAEDs comedication or compensated by administering higher imatinib doses. In addition these data demonstrate that there is no significant difference in the pharmacokinetics of imatinib between patients with glioblastoma and CML.

## INTRODUCTION

Glioblastoma multiforme (GBM) is the most frequent primary malignant intracranial tumor in adults. Despite advances in multidisciplinary treatment approaches like neurosurgery, radiation and chemotherapy the overall prognosis remains poor with a median survival between 12 and 18 months after diagnosis. Recurrence under optimal treatment including chemotherapy with temozolomide seems to be nearly unavoidable. Following progression the available therapies like surgical reintervention, external beam radiotherapy and salvage chemotherapy provide a limited survival benefit [1-6].

In search of new treatment strategies preclinical and clinical research focussed on novel agents targeting distinct signaling pathways. CNS malignancies including GBM were found to express epithelial growth factor-receptor (EGF-R) and platelet derived growth factor-receptor (PDGF-R). Imatinib mesylate (STI571, Gleevec) is a selective inhibitor of the tyrosine kinases BCR-ABL, c-KIT and PDGF-R and has been shown to be highly active in chronic myeloid leukemia (CML) and has significant antitumor efficacy against gastrointestinal stroma tumors (GIST). In GBM only modest responses could be reached if imatinib is used as a single agent. In contrast, a combination of 600mg imatinib with 1000mg Hydroxyurea (HU) daily leads to an increased response rate in patients with progressive GBM. Currently, the mechanism underlying the enhanced activity of this combination regime is not well understood [7-14].

Concomitant administration of enzyme-inducing antiepileptic drugs (EIAEDs) as well as non enzyme-inducing antiepileptic drugs (non-EIAEDs) are a common therapy in patients with brain tumors. The EIAEDs carbamazepine and phenytoin are potent inducers of cytochrome P450 isoenzyme CYP3A4, whereas oxcarbazepine and topiramate are known as weak inducers of the enzyme. The non-EIAEDs levetiracetam and maybe lamotrigine are most likely not involved in drug interactions. Valproic acid slightly inhibits CYP3A4 activity and is able to significantly displace drugs from plasma albumin. Imatinib is metabolised mainly by cytochrome P450 CYP3A4 to its main metabolite CGP74588 that has similar *in vitro *activity to the parent compound. Therefore significant interactions may occur between imatinib and EIAEDs leading to changes in plasma concentration of imatinib, CGP74588 as well as coadministered drugs. Some studies have investigated the effects of EIAEDs on the pharmacokinetics of imatinib and CGP74588 [15-21, 25]. They demonstrated that EIAED’s could lead to a substantially decreased plasma exposure of imatinib. In patients receiving imatinib for chronic myelogenous leukemia and gastrointestinal stromal tumors they should be avoided if possible. On the other hand, imatinib is still the most important drug for the therapy of CML and GIST, and moreover the pk of alternative tyrosine kinase inhibitors is presumably also altered by EIAEDs.

To improve the knowledge about the influence of the various EIAEDs on the imatinib pk, we determined trough levels of imatinib and CGP74588 in a collective of 224 GBM patients. The analysed imatinib trough level confirm the results that were found in earlier studies and in addition demonstrate, that the imatinib pk from GBM patients without EIAED application is not different from the pk in CML patients.

## PATIENTS AND METHODS

### Patients and Sample Collection

We analyzed trough levels of imatinib and CGP74588 in a total of 224 patients (age 19 to 69 years, median 51 years) with histologically confirmed diagnosis of glioblastoma multiforme/ astrocytome WHO grade IV who received 300 - 600mg imatinib o.d. (once daily) and 2 - 3 x 500mg Hydroxyurea p.o. daily. The differences of the median age and gender in the compared trough level groups were not significant. All presented trough levels were linearly calculated for a 600mg / o.d. dose of imatinib. Group A consists of 111 patients treated with imatinib and HU without antiepileptic co-medication (non-AED), Group B consists of 28 patients treated with imatinib and HU in combination with non-EIAEDs, Group Cconsists of 85 patients treated with imatinib and HU in combination with EIAEDs. In group B (non-EIAED) 4 patients received lamotrigine, 15 valproic acid and 9 levetiracetam. In group C (EIAED) 15 patients received phenytoin, 63 carbamazepine, 6 oxcarbazepine and 1 topiramate. Patients who were concomitantly treated with other CYP3A4 inducing drug compounds were excluded. At the beginning of treatment all patients had adequate renal (serum creatinine ≤ 1.5 x ULN), hepatic (SGOT and SGPT ≤ 2.5 x ULN, total bilirubin ≤ 1,5 x ULN) and bone marrow (ANC ≥1.5 x 109/L, platelets ≥ 100 x 109/L and Hgb >10g/dL) function.

Blood samples were scheduled on week 2, 4, 6, 18, 30 and 42 of the study prior to the morning dosage administration, but only in a small portion of patients all scheduled samples were drawn in this multicenter study (543 samples from 224 patients). Samples were centrifuged at 1000g for 10min, 1ml of plasma was separated, stored at –20°C and sent by express mail to our laboratory for analysis.

### HPLC Measurement

The concentration of Imatinib and CGP74588 was determined using a single high performance liquid chromatograph method with ultraviolet detection. After protein precipitation samples were prepared and both substances were online enriched on a Zirchrom-PBD (ZirChrom Separations, USA, Anoka) guard column. Analysis was performed with a Zirchrom-PBD analytical column followed by ultraviolett detection at 260nm. Lower limit of quantification was 10ng/ml for both Imatinib and CGP74588. The intra-day precision in plasma samples, as expressed by the coefficient of variation, ranges between 1.74% and 8.60% for imatinib and 1.45% and 8.87% for CGP74588, depending on the concentration. The inter-day precision for a plasma concentration of 1000 ng/ml analyzed over a 7-month time period was 8.31% for imatinib and 6.88% for CGP74588.

The method was validated and is established for routine use as described previously [22-24]. The GINA star software (Raytest, Straubenhardt, Germany) was used for data acquisition, evaluation and integration of chromatograms.

### Pharmacokinetics

All trough concentrations of imatinib and CGP74588 were determined after the patients had received imatinib for at least two weeks. In consideration of the half- life of imatinib (16.5- 26h) and CGP74588 (29,5- 73,5h) within this period all patients should have reached pseudo steady state conditions [23-25]. The obtained data were compared between treatment groups, and with historical concentrations and simulations from CML patients (Reference). The mean, median, maximum (max), minimum (min), standard deviation (SD) and coefficients of variation (CV) were calculated for the trough concentrations using the Microsoft excel software.

### Statistical Analysis

The statistical analysis was performed using Excel and SPSS for Windows. Comparisons between groups were perfomed using a t-test for independent samples. The resulting trough levels are presented as means, standard deviation (±SD) and coefficient of variation (CV), if not otherwise indicated. Differences were considered as statistically significant if a P-value < 0.05 was achieved.

## RESULTS

The results of the measured imatinib and CGP74588 steady-state trough concentrations in 543 plasma samples from the enrolled 224 GBM patients are summarized in tables 1 and 2 for a dose of 600mg imatinib o.d. No significant differences in trough level of imatinib were observed between group A (non-AED) C_trough_ = 1404 ng/ml (CV 64%) and group B (non-EIAED) C_trough_ = 1374 ng/ml (CV 46%). Analogous results were found for CGP74588, group A C_trough_ = 356 ng/ml (CV 52%) and group B (non-EIAED) C_trough_ = 351 ng/ml (CV 34%). The parameters which were obtained previously from hematological patients, C_trough_ = 1400ng/ml (CV 50%) for imatinib and C_trough_ = 300ng/ml (CV 50%) for CGP74588 were quite similar to those seen in groups A and B. Even within group B the comparison of separate drug compounds levetiracetam (imatinib: C_trough_ = 1369 ng/ml,CV 47%; CGP74588: C_trough_ = 347 ng/ml, CV 36%), lamotrigine (imatinib: C_trough_ = 1466ng/ml, CV 28%; CGP74588: C_trough_ = 431ng/ml, CV 25%) and valproic acid (imatinib: C_trough_ = 1399ng/ml, CV 47%; CGP74588: C_trough_ = 355ng/ ml, CV 33%) showed no significant difference.

The patients treated with EIAEDs (group C) showed a significant decrease of about 68% in the steady-state level of Imatinib C_trough_ = 477 ng/ml (CV 70%, P<0.05) compared to the other groups. Furthermore the values within group C had a greater variability as compared to groups A and B. Patients receiving phenytoin showed the lowest concentrations of imatinib: C_trough_ = 380 ng/ml (CV 70%), followed by C_trough_ = 473ng/ml (CV 76%) for patients on carbamazepine. In the oxcarbazepine group we obtained a C_trough_ = 534 ng/ml (CV 36%) and from one patient taking topiramate four measured plasma samples gave C_trough_ = 722ng/ml (CV 28%). Thus the decrease in imatinib trough level in group C ranged from about 73% to about 48% compared to groups A and B. In contrast the influence of EIAEDs on metabolite were less remarkable but although significant. For CGP74588 a mean C_trough_ = 240 ng/ml (CV 59%, P<0.05) was found corresponding to a decrease of about 32% compared to group A and B. Within group C for phenytoin (C_trough_ = 268 ng/ml CV 73%), carbamazepine (C_trough_ = 240 ng/ml CV 57%) and oxcarbazepine (C_trough_ = 216 ng/ml CV 40%) quite similar values were found. Only the patient on topiramate showed a slightly higher CGP74588 level C_trough_ = 291ng/ml CV 48%.

**Fig. (1):**
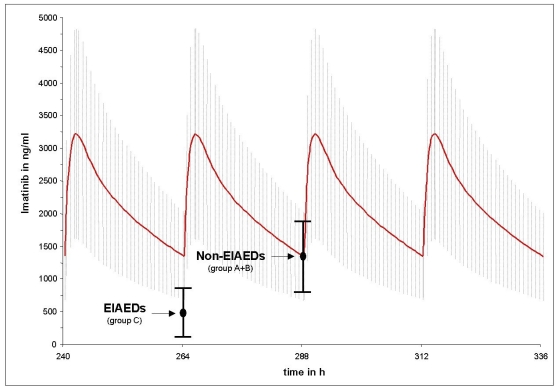
Differences between the mean trough level of imatinib in patients on EIAEDs and without EIAEDs compared to the mean imatinib trough level in CML patients without antiepileptic drugs. Points indicate mean measured trough level of imatinib and black vertical bars indicate the standard deviation. The red curve represents the calculated mean imatinib plasma decay curve under pseudo steady-state conditions from CML patients taking 600mg imatinib o.d.. The grey hutching represents the related standard deviation.

**Fig. (2):**
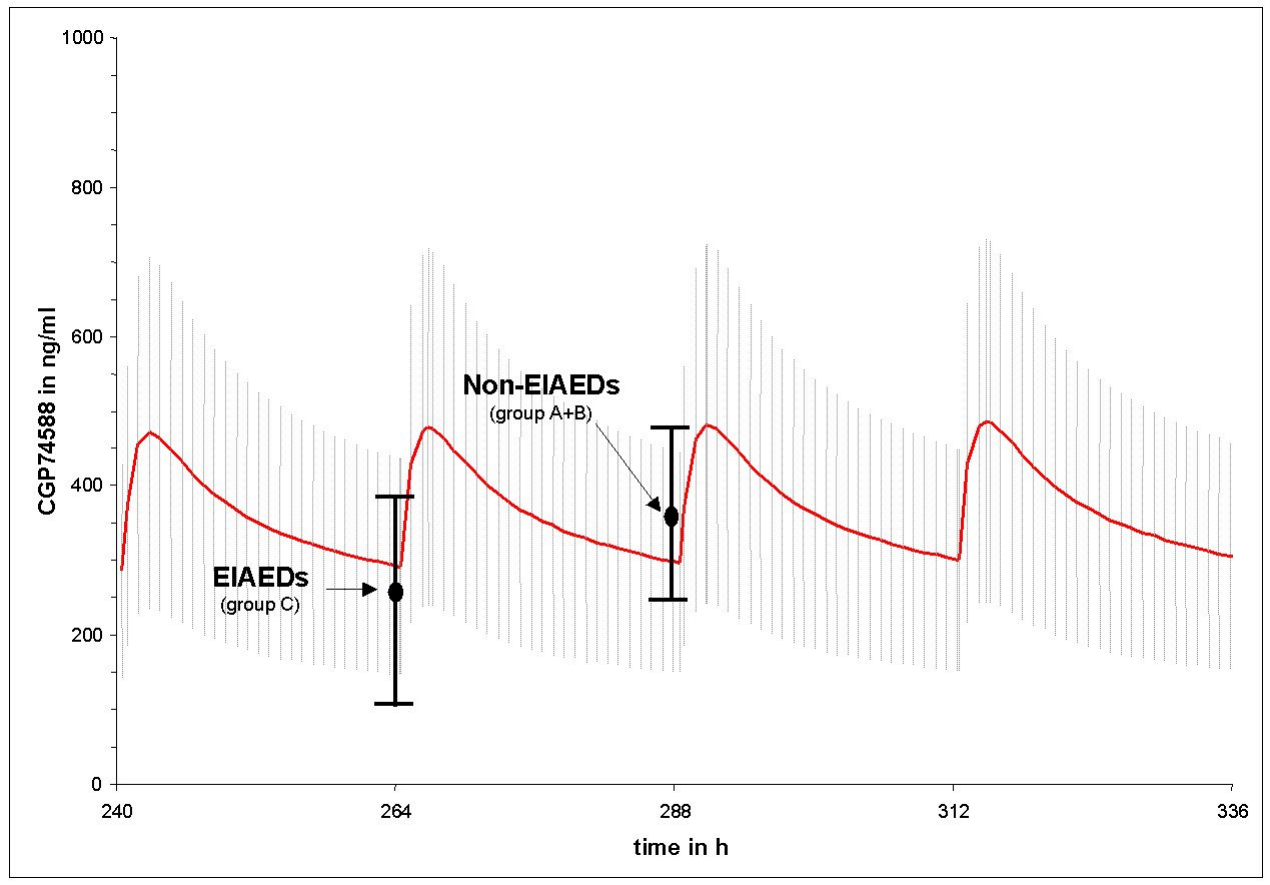
Differences between the mean trough level of CGP74588 in patients on EIAEDs and without EIAEDs compared to the mean of CGP74588 trough level in CML patients without antiepileptic drugs. Points indicate mean measured trough level of CGP74588 and black vertical bars indicate the standard deviation. The red curve represents the calculated mean CGP74588 plasma decay curve under pseudo steady-state conditions from CML patients taking 600mg imatinib o.d.. The grey hutching represents the related standard deviation.

Figs. (1 and 2) compares the mean imatinib and CGP74588 trough level results of group A (non-AED) plus B (non-EIAEDs) to group C (EIAEDs), depicted on the calculated plasma curves from CML patients. They show that the mean trough level of group A plus B fits nearly perfectly to the the CML curves, while for group C significant decreases were observed.

The mean CGP74588 to imatinib concentration ratios for groups A, B and for patients with CML were 25%, 26% and 21%, respectively. Due to the higher decrease of imatinib concentrations compared to CGP74588 the ratio in patients on EIAEDs was high, 50%.

## DISCUSSION

In the present investigation, EIAED- treated patients displayed a significantly reduced trough level of imatinib in comparison with patients who were on non-EIAEDs or did not take AEDs, respectively. The respective differences were less pronounced but although significant for CGP74588. In a recent publication, Wen *et al*. evaluated plasma concentrations of imatinib and CGP74588 in 11 patients that were concomitantly treated with EIAEDs and the same number of patients not receiving EIAEDs. Results demonstrate a decrease of imatinib plasma AUC of about 70% and of CGP74588 plasma AUC of about 10%, respectively. The overall AUC of imatinib and CGP74588 was reduced 2.7 fold whereas the calculated trough concentration C_min_ showed a decrease of about 79% for imatinib and of about 40% for CGP74588 [20]. Table **3** compares these findings to our study. The results of this investigation are in line with our findings of 68% (about 2.9 fold) and 32% decline in trough levels of imatinib and CGP74588 in patients receciving EIAEDs. Additionally, our results are confirmed by the report of Reardon *et al.*, where 17 patients with GBM were treated with imatinib and HU. The patients (n=8), who were not receiving EIAEDs had pharmacokinetic values comparable to those from patients with haematological malignancies and GIST. In contrast, patients (n=9) receiving EIAEDs showed a significant shorter t_1/2_, a lower AUC and higher Cl_app_ than the other group. The influence of EIAEDs on the pharmacokinetics of CGP74588 was only moderate [15]. Similar effects were seen after pretreatment with the CYP3A4 inductor rifampicin in healthy volunteers with a 70% decrease of the AUC of imatinib. The slight increase in the concentration of CGP74588 observed in that report might be explained by the fact that patients had received only two single doses of imatinib within two weeks and therefore had not reached steady-state levels of imatinib and CGP74588 [21].

Within the group of EIAEDs, the effects of phenytoin and carbamazepine on imatinib trough levels were comparable. As expected, thee effect of oxcarbazepine, a weaker CYP3A4 inducer on imatinib exposure was less profound. The patient receiving topiramate showed a less prominent decrease of imatinib concentrations (48%) compared to the rest of the EIAED group (68%) [17]. Since the data on topiramate-imatinib interaction are derived from one patient only, this result has to be interpreted with caution. But still, the observation would fit very well to the less pronounced enzyme-inducing activity reported for topiramat. These results suggest that a considerable increase of the individual imatinib dose may be considered in patients on EIAEDs. Reardon *et al*. reported that in patients with GBM on EIAEDs who received HU and 1000 mg imatinib, the imatinib exposure remained significantly lower compared to patients on non-EIAEDs. Surprisingly, patients with EIAEDs intake had an improved progression free survival. On the other hand, in spite of the lower AUC of imatinib the observed toxicity was significant which lead to a maximum tolerated dose of 500mg imatinib twice daily for patients with EIAED co-medication [15]. However, patients with CML or GIST who receive imatinib and EIAEDs will be at an increased risk of not being able to achieve the optimal clinical response due to a decreased imatinib exposure. On the other hand, patients whose dose is to be escalated should be carefully monitored for signs of toxicity or intolerance.

In summary, we observed a significant decrease of imatinib and CGP74588 exposure in patients taking EIAEDs. No significant difference in imatinib trough concentrations was observed between GBM patients who did not receive AEDs or non-EIAEDs and CML patients. For the compounds levetiracetam, lamotrigine and even valproic acid, the later being considered as a weak inhibitor of CYP3A4 [19], we found no effect on imatinib trough levels. Thus, switching to non-EIAEDs if anticonvulsive therapy is needed, might be an option for patients receiving imatinib treatment. Application of Imatinib and EIAEDs should only done if absolutely necessary, and if so in combination with repetitively trough level controls.

## Figures and Tables

**Table 1:** Mean Trough Levels and Statistical Parameters for Imatinib Mesylate and CPG74588 in GBM Patients (All Data refer to a 600mg Imatinib o.d. Application)

	Group A no Antiepileptics	Group B Non EIAED Antiepileptics		Group C EIAED Antiepileptics			not Clearly Classified	
		Valproic Acid	Levetiracetam	Phenytoin	Carbamazepine	Oxcarbazepine	Topiramate	Lamotrigine
**n samples**	224	28	24	22	199	33	4	9
**n patients**	111	15	9	15	63	6	1	4
	**imatinib trough level in ng/ml**							
**Mean ng/ml**	1404	1399	1369	380	473	534	722	1466
**SD ng/ml**	899	664	640	266	358	193	199	405
**CV in %**	64	47	47	70	76	36	28	28
**Median in ng/ml**	1245	1264	1206	390	363	569	724	1249
**Max in ng/ml**	6881	3368	2678	1001	1952	868	960	2019
**Min in ng/ml**	38	235	383	22	14	127	481	1050
	**CGP74588 trough level in ng/ml **							
**Mean ng/ml**	356	355	347	268	240	216	291	431
**SD ng/ml**	186	117	123	196	137	86	140	107
**CV in %**	52	33	36	73	57	40	48	25
**Median in ng/ml**	323	311	338	205	209	204	297	420
**Max in ng/ml**	1482	616	663	768	724	464	455	577
**Min in ng/ml**	27	192	170	45	40	50	114	278

**Table 2:** Mean Trough Levels in Three GBM Patient Groups and in CML Patients. All Data refer to a 600mg Imatinib o.d. Application

	Group A no-AED	Group B non-EIAED	Group C EIAED	CML Patients
	imatinib trough level in ng/ml			
**mean in ng/ml**	1404	1374	477	1400
**SD in ng/ml**	899	631	335	700
**CV in %**	64	46	70	50
	**CGP74588 trough level in ng/ml**			
**mean in ng/ml**	356	351	240	300
**SD in ng/ml**	186	121	137	150
**CV in %**	52	34	57	50
	**Ratio CGP74588/imatinib in %**			
**mean in %**	25	26	46	21

**Table 3:** Dose-Normalized Trough Levels Published by Wen *et al*. Compared with the Data in the Present Study

	Wen *et al*. 2006 [20]		Present Study			
Dose-normalized C_min/through_ (ng/ml/mg) at steady state	non-EIAED	EIAED	no-AED	non-EIAED	EIAED	CML Patients
**Mean imatinib**	2.8	0.58	2.34	2.29	0.80	2.33
**Mean CPG74588**	0.70	0.42	0.59	0.59	0.40	0.50
**Mean CPG74588/imatinib ratio**	0.25	0.71	0.26	0.26	0.50	0.21
